# Enhanced dispersal capacity in edge population individuals of a rapidly expanding butterfly

**DOI:** 10.1002/ece3.10885

**Published:** 2024-02-01

**Authors:** Anaïs Dederichs, Klaus Fischer, Peter Michalik, Michaël Beaulieu

**Affiliations:** ^1^ Zoological Institute and Museum University of Greifswald Greifswald Germany; ^2^ Institute for Integrated Sciences University of Koblenz Koblenz Germany

**Keywords:** dispersal syndrome, flight, metabolic rate, range expansion

## Abstract

Natural range shifts offer the opportunity to study the phenotypic and genetic changes contributing to colonization success. The recent range shift of the Southern small white butterfly (*Pieris mannii*) from the South to the North of Europe offers a prime example to examine a potential dispersal syndrome in range‐expanding individuals. We compared butterflies from the core and edge populations using a multimodal approach addressing behavioral, physiological, and morphological traits related to dispersal capacity. Relative to individuals from the core range (France), individuals from the edge (Germany) showed a higher capacity and motivation to fly, and a higher flight metabolic rate. They were also smaller, which may enhance their flight maneuverability and help them cope with limited resource availability, thereby increasing their settlement success in novel environments. Altogether, the behavioral, physiological, and morphological differences observed between core and edge populations in *P. mannii* suggest the existence of a dispersal syndrome in range‐expanding individuals. Whether these differences result from genetic and/or phenotypic responses remains, however, to be determined.

## Graphical abstract

We investigated differences in flight performances between core and edge population of the Southern small white butterflies (*Pieris mannii*) currently expanding its range northward. We found higher movement capacity in the individuals from the newly colonized range suggesting a potential dispersal syndrome in the edge population, which likely contributed to the rapid range expansion of this species.

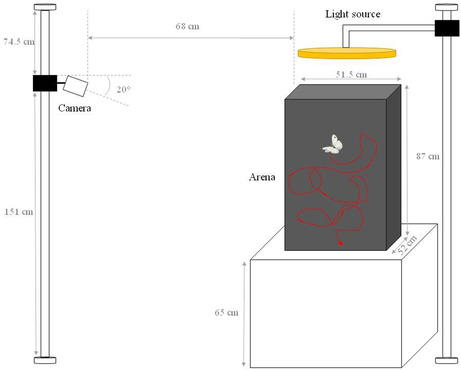

## 1 INTRODUCTION

To cope with adverse conditions, organisms may use phenotypic plasticity (Rodrigues & Beldade, 2020) or dispersal to more beneficial places (Berg et al., 2010). The specific coping response presumably depends on the relative cost/benefit ratio associated with each strategy, the existence of milder places and the capacity of organism to move. For instance, sessile organisms are more likely to use phenotypic plasticity, while highly mobile ones are more likely to move. Accordingly, butterflies often track climate changes by shifting their range in latitude and/or altitude (Chen et al., 2011; Parmesan et al., 1999; Rödder et al., 2021). This capacity to track climate changes is likely related to different factors underlying movement (Le Roy et al., 2019), including behavior, physiology, and morphology (Flockhart et al., 2017; Mitikka & Hanski, 2009; Tsai et al., 2020), jointly determining the cost–benefit ratio of dispersal (Renault, 2020).

Because of the novel environmental conditions encountered at the expansion range, a quick fixation of traits facilitating expansion is expected due to eco‐evolutionary feedback mechanisms (Armsworth & Roughgarden, 2005; Boeye et al., 2013; Hill et al., 2011; Pierce et al., 2014; Schrieber & Lachmuth, 2017), which in turn may result in the appearance of dispersal syndromes (Alex et al., 2013; Doebeli & Ruxton, 1997; Saastamoinen et al., 2012; Shine et al., 2011; Thomas et al., 2001) (i.e., a set of correlated behavioral, physiological, and morphological traits facilitating dispersal) (Cote et al., 2010; Legrand et al., 2016; Saastamoinen et al., 2012). This scenario possibly applies to the Southern small white butterfly (*Pieris mannii*), originally mainly restricted to the Mediterranean Basin, but currently expanding to the North of Europe with an unparalleled speed (Feldtrauer & Feldtrauer, 2009; Meineke & Menge, 2016; Vantieghem, 2018; Wiemers, 2016). The use of new host plants and a faster growth rate at higher latitudes have likely been contributing to this expansion success (Neu et al., 2021; Neu & Fischer, 2022). However, whether a dispersal syndrome is also contributing to this expansion remains unknown. Therefore, we compared here behavioral, physiological, and morphological traits related to dispersal ability in *P. mannii* butterflies from core and edge populations. We predicted a higher dispersal ability in individuals from the newly colonized range reflecting selection for a specific phenotypic design.

## 2 MATERIALS AND METHODS

In July 2020, adult *P. mannii* females were collected from the species' core distribution area (southern France: Valbonne 43.63° N 7.02° E, *N* = 11) and the newly colonized range (Germany: Verl 51.87° N 8.52° E, *N* = 13), and were transferred to a climate chamber for egg‐laying at the University of Greifswald, Germany (23°C, 60% relative humidity, L20:D4). Females were kept in translucent boxes (30 × 20 × 21 cm), each containing a *Diplotaxis tenuifolia* leaf for oviposition, fresh flowers, 20 vol. % sucrose solution, and water for feeding. Eggs were collected and leaves replaced daily. Under the same controlled conditions as adult females experienced (23°C, 60% relative humidity, L20:D4), hatched larvae were reared individually in translucent plastic boxes (4 × 10 × 6 cm) with moistened filter paper and ad libitum access to fresh cuttings of *Brassica napus oleifera* until pupation.

**FIGURE 1:**
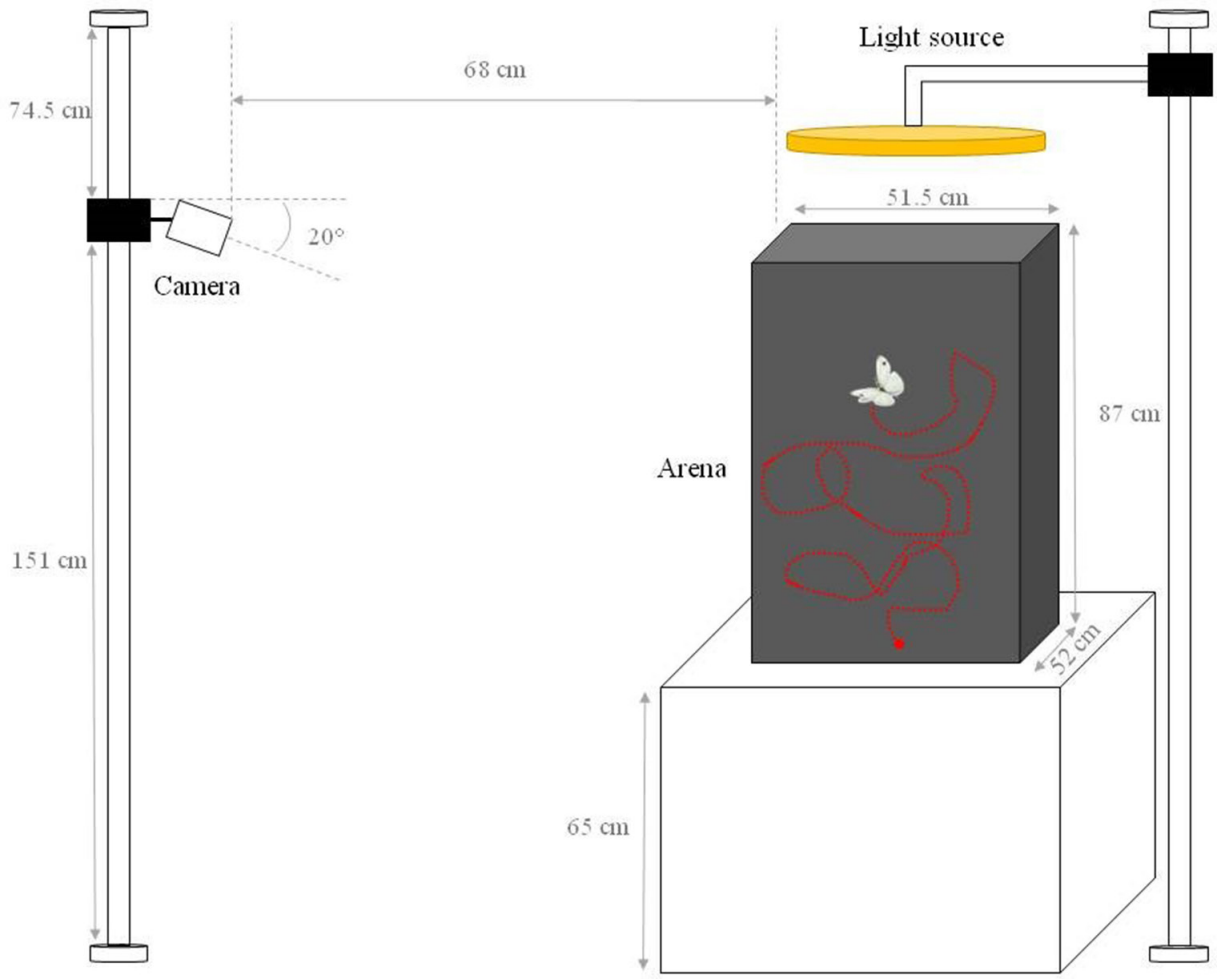
Experimental setup for flight tracking.

After eclosion, 8 females and 8 males from each country (*N*_total_ = 32 individuals) were individually marked. One‐day old individuals were weighed to the nearest 0.01 mg, before being immediately tested for flight parameters. All individuals were tested for flight willingness (arena test) and capacity (shaker test) under the same conditions as used for rearing. For scoring flight willingness, butterflies were released in a black arena (87 × 52 × 51.5 cm) with a full‐spectrum light source at the top (FalconEyes, DVR‐300DVC, Hong Kong, China), and were stimulated to fly by gently touching them with plastic tweezers (Figure 1). Between 6 and 17 flights per individual were recorded by a camera (Basler ace, Ahrensburg, Germany) placed in front of the arena. Flight duration, flight distance and maximal velocity were then calculated for each flight using EthoVision XT 14 (Noldus IT, Leesburg, The Netherlands).

In a second step, flight endurance and metabolic rate were simultaneously measured using a ‘shaker test’ during which butterflies were placed into a sealed 1‐L plastic chamber coupled with a stop‐flow respirometry system (Q‐bit system, Q‐S151 C0_2_ Analyzer, Kingston, Canada). After 5 min, CO_2_ production (“resting metabolic rate”) was measured via a 5 min stop/10 min flush cycle using “Logger Pro” (v. 3.15, Vernier, Beaverton, Oregon, USA) with dried air flowing through at a rate of 130–140 mL/min during the flushing period. Then, to measure movement capacity (time spent flying) and flight metabolic rate (CO_2_ production during flight), butterflies were forced to fly during the 5 min of the stop flow cycle by strongly shaking the chamber using a rapid agitator (Ducatez et al., 2012). Following flight measurements, butterflies were frozen and stored at −80°C for morphological analyses.

Frozen butterflies were first weighed (±0.01 mg) before removing their wings, head and legs on dry ice. Their thorax and abdomen were then separated and weighed separately (±0.01 mg). The fore‐ and hindwings (dorsal and ventral side) were photographed under standardized light conditions with a digital camera (Canon EOS 500D equipped with an Ultrasonic Sigma 100 mm macro lens) mounted on a focal‐plane adjustable stand. The length of the forewings (from basis to apex) and the area of the fore‐ and hindwings were measured using the lasso tool in Adobe Photoshop CS (Adobe, Inc., San José, CA, USA). Wing loading was calculated as total body mass divided by total wing area (forewings + hindwings), and the wing aspect ratio of forewings as $\frac{4\times {\text{wing length}}^{2}}{\text{wing area}}$ to describe wing shape (higher values reflecting more slender wings [Betts & Wootton, 1988]). The thorax–abdomen ratio was calculated by dividing thorax mass by total mass to reflect the relative investment in thorax flight muscles.

Because no statistical difference was found for any of the measured parameters between males and females (all *p* > .05), we pooled both sexes for further statistical analyses. Statistical comparisons between core and edge populations were performed via a Mann–Whitney‐Wilcoxon *U*‐test for non‐repeated measurements. For repeated measurements (flight distance, duration (log transformed), and maximal velocity), linear mixed models (package “lme4”, v1.1.27.1 [Bates et al., 2015]) were used with individuals as a random factor (the “ranova” function from the “lmerTest” package, v3.1.3 (Kuznetsova et al., 2017) was used to calculate *p*‐values associated with the random effect). Results are provided as means ± SE for each experimental group in Table 1. Finally, correlations between physiological, morphological and flight parameters were calculated via the “rcorr()” function of the “Hmisc” package, v4.6.0 (Frank & Harrell, 2021). All statistical analyses were performed in R (v4.1.2; R Core Team, 2021).

**TABLE 1:** Results of linear mixed models and Mann–Whitney *U*‐test for differences in physiological and morphological parameters between the core and edge population of *Pieris mannii*.

	Origin	Individual (random)
Flight duration	*χ* ^2^(1) = 12.5, ***p* < .001**	*χ* ^2^(1) = 39.4, ***p* < .001**
Flight distance	*χ* ^2^(1) = 18.7, ***p* < .001**	*χ* ^2^(1) = 29.2, ***p* < .001**
Maximal velocity	*χ* ^2^(1) = 10.2, ***p* = .002**	*χ* ^2^(1) = 5.6, ***p* = .018**
Flight endurance	*W* = 48, ***p* = .007**	
Total wet mass	*W* = 167, *p* = .149	
Flight metabolic rate	*W* = 58, ***p* = .0424**	
Resting metabolic rate	*W* = 123, *p* = .683	
Forewing length	*W* = 224.5, ***p* < .001**	
Wing loading	*W* = 134, *p* = .838	
Wing aspect ratio	*W* = 56, ***p* = .006**	
Thorax–abdomen ratio	*W* = 185, ***p* = .032**	

*Note*: Significant *p*‐values (*p* < .05) are shown in bold.

## 3 RESULTS

**FIGURE 2:**
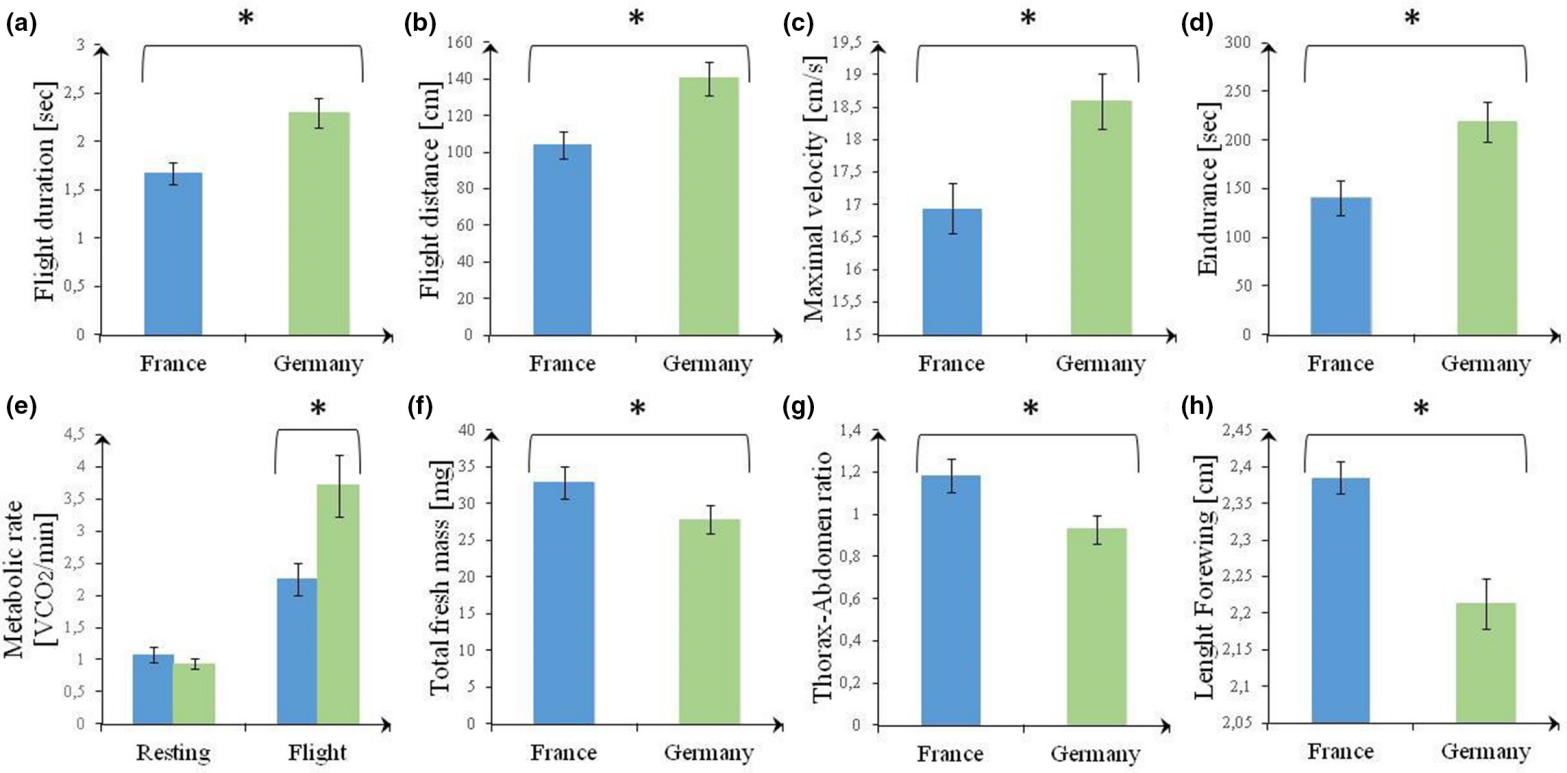
The flight duration (a), flight distance (b), maximal velocity during flight (c), flight endurance (d), metabolic rate (e), total fresh mass (f), thorax–abdomen ratio (g), and the forewing length (h) of *Pieris mannii* from France and Germany (means ± SE). *A statistically significant difference between both populations.

Individuals from the edge population flew in the arena significantly longer, further, and with a greater maximum velocity than butterflies from the core population (Table 1, Figure 2). Additionally, butterflies from the edge population were more endurant in the shaker test. While resting metabolic rate did not differ between both populations, butterflies from the edge population had a higher flight metabolic rate than those from the core population. Except for wing loading, all morphological parameters differed between both populations, with butterflies from the edge population having a lower mass, smaller wings, and lower thorax–abdomen ratio. Very few significant correlations were found between morphology and flight parameters, the most meaningful ones being: in the core population, (1) a negative correlation between wing loading and flight metabolic rate (*r*_s_ = −.61, *p* = .047); (2) a positive correlation between total wet mass and wing aspect ratio and in the edge population (3) a negative correlation between total fresh mass and flight duration (*r*_s_ = −.70, *p* = .024); (4) a positive correlation between total fresh mass and flight distance (*r*_s_ = .74, *p* = .015), and (5) a positive correlation between wing aspect ratio and flight duration (*r*_s_ = .64, *p* = .048; Tables S1 and S2).

## 4 DISCUSSION

We found strong behavioral, physiological and morphological differences between butterflies from the edge and the core populations of *Pieris mannii*, which may be contributing to its rapid expansion. For instance, the longer flights made by butterflies from the edge population in the arena may reflect a higher exploratory propensity (Cote et al., 2010; Ducatez et al., 2012). However, the fact that these butterflies also fly longer when forced to fly in the shaker test additionally suggests that they have a higher flight capacity. Similarly, flight willingness and capacity were previously found to correlate positively in *Lycaena tityrus* butterflies monitored in controlled environments (Reim et al., 2019). However, while such behavioral differences between edge and core populations as measured in the lab might be used in predictive models estimating metapopulation dynamics (Ovaskainen & Hanski, 2004), how they actually translate into dispersal differences under natural conditions remains unknown (Stevens et al., 2010). Further studies conducted under semi‐natural conditions are therefore necessary to examine the connection between laboratory flight behavior and dispersal in the field (Cote et al., 2022; Legrand et al., 2015; Reim et al., 2018).

Flight ability (and hence dispersal ability) directly depends on flight metabolic rate (Haag et al., 2005; Hanski et al., 2004; Niitepõld et al., 2009; Rauhamäki et al., 2014). The higher flight metabolic rate measured in butterflies from the edge population could be seen as reflecting higher flight costs (thereby counterintuitively limiting the flight capacity of expanding individuals), but also alternatively a higher capacity to generate energy to sustain flights (thereby facilitating the flight capacity of expanding individuals). Perhaps, in agreement with this second hypothesis, the higher metabolic plasticity of these butterflies between resting and flying may underlie their better flight performance (Mattila, 2015; Van Dyck & Holveck, 2016).

Wings with a shape that minimizes flight costs are usually assumed to enhance dispersal ability. For instance, individuals with large wings facilitating gliding and/or with wings with a higher aspect ratio creating less drag are usually assumed to be the most dispersive (Ancel et al., 2017; Dudley, 2002; Le Roy et al., 2019; Park et al., 2010). Even though the wing aspect ratio and flight duration positively correlated in individuals from the edge population, these butterflies also had smaller wings than those from the core population. The higher aspect ratio combined with the smaller wing size of individuals from the edge population may optimize the balance between long distance flight capability and maneuverability and hence enhance their exploring capacity in novel environments. This hypothesis is confirmed by the negative correlation we found between mass and flight duration (i.e., smaller individuals being more endurant) and seems to reveal a potential selection for specific physical characteristics linked to flight. On the other hand, the positive correlation found in the core population between mass and wing aspect ratio exhibits an opposite form of selection on the physical characteristic of the individuals, overall, influencing less the flight parameters. Finally, the lower thorax–abdomen ratio observed in butterflies from the edge population appears counter‐intuitive, as it is usually related to a lower flight performance (Chang et al., 2021; Langellotto et al., 2000). This relatively smaller thorax (as well as their overall smaller size) may be a side effect of a faster development (Neu & Fischer, 2022), and may help individuals from the edge population to cope with suboptimal resources (smaller individuals having lower energetic requirements). However, despite this apparent lower investment in flight muscles, butterflies from the edge population were still able to fly longer than those from the core population, which may result from physiological adjustments including metabolic changes (potentially related to mitochondrial efficiency [Toews et al., 2014]).

Behavioral, physiological, and morphological mechanisms facilitating range shift potentially reflect both genetic and plastic responses. In our study, it was not possible to distinguish between the contribution of both responses in the differences we observed between the core and the edge populations. Even though the recent expansion of *Pieris mannii* may suggest a higher contribution of phenotypic plasticity, genetic changes still seem possible (as suggested for body size and development time [Neu & Fischer, 2022]). Irrespective of the underlying mechanisms, our study strongly suggests the existence of a dispersal syndrome in *P. mannii* contributing to its northwards expansion. However, even though dispersal is an essential part of the expansion process, dispersing individuals must also survive and successfully reproduce in their new environment. Here, host shifts and life‐history adaptations may play an important role (Neu et al., 2021; Neu & Fischer, 2022). Thus, to fully understand the factors underlying range expansions, integrated studies including the dispersal, life history and resource use are much needed.

## AUTHOR CONTRIBUTIONS

**Anaïs Dederichs:** Conceptualization (equal); data curation (lead); formal analysis (lead); investigation (equal); methodology (lead); project administration (supporting); validation (equal); visualization (lead); writing – original draft (lead); writing – review and editing (equal). **Klaus Fischer:** Funding acquisition (lead); investigation (supporting); methodology (supporting); validation (equal); writing – review and editing (equal). **Peter Michalik:** Conceptualization (equal); funding acquisition (lead); investigation (supporting); methodology (supporting); project administration (lead); resources (lead); software (lead); supervision (equal); validation (equal); visualization (equal); writing – review and editing (equal). **Michaël Beaulieu:** Conceptualization (equal); investigation (equal); methodology (equal); supervision (equal); validation (equal); visualization (equal); writing – review and editing (equal).

## CONFLICT OF INTEREST STATEMENT

The authors declare that they have no competing interests.

## Supporting information


Table S1.
Table S2.

## Data Availability

The data are provided in the electronic supplementary material.
