# Supplementary material for: Enhanced dispersal capacity in edge population individuals of a rapidly expanding butterfly
Source: Ecol Evol. 2024 Feb 1;14(2):e10885. doi: 10.1002/ece3.10885 (PMC10834214; doi:10.1002/ece3.10885)
Supplement: Supplementary file 1 — Table S1. Table S2. [file ECE3-14-e10885-s001.docx]

**Supplementary Tables**

| Flight Duration | **r_s_ = 0.939,**  **P < 0.001 *** | r_s_ = 0.461,  P = 0.154 | r_s_ = -0.22,  P = 0.517 | r_s_ = 0.559,  P = 0.074 | \| r_s_ = -0.142,  P = 0.677 \| \| --- \| \| rs = -0.161,  P = 0.637 \| \| rs = -0.073,  P = 0.831 \| \| rs = 0.435,  P = 0.182 \| \| rs < 0.001,  P = 0.999 \| \| Flight metabolic Rate \| \| rs = -0.368,  P = 0.265 \| \| rs = -0.602,  P = 0.05 \| \| rs = 0.172,  P = 0.613 \| \| rs = 0.269,  P = 0.423 \| | r_s_ = 0.468,  P = 0.147 | r_s_ = -0.117,  P = 0.731 | r_s_ = 0.554,  P = 0.077 | r_s_ = -0.187,  P = 0.581 |
| --- | --- | --- | --- | --- | --- | --- | --- | --- | --- | --- | --- | --- | --- | --- | --- | --- | --- | --- | --- |
| **rs = 0.939,**  **P < 0.001 *** | Flight Distance | r_s_ = 0.153,  P = 0.653 | r_s_ = 0.239,  P = 0.479 | r_s_ = 0.417,  P = 0.202 | r_s_ = -0.161,  P = 0.637 | r_s_ = 0.45,  P = 0.165 | r_s_ = -0.033,  P = 0.924 | r_s_ = 0.457,  P = 0.157 | r_s_ = -0.2,  P = 0.556 |
| rs = 0.461,  P = 0.154 | rs = 0.153,  P = 0.653 | Maximal Velocity | r_s_ = -0.091,  P = 0.791 | r_s_ = 0.376,  P = 0.254 | r_s_ = -0.073,  P = 0.831 | r_s_ = 0.193,  P = 0.569 | r_s_ = -0.307,  P = 0.358 | r_s_ = 0.286,  P = 0.393 | r_s_ = 0.12,  P = 0.973 |
| rs = -0.22,  P = 0.517 | rs = 0.239,  P = 0.479 | rs = -0.091,  P = 0.791 | Flight endurance | r_s_ = -0.14,  P = 0.681 | r_s_ = 0.435,  P = 0.182 | r_s_ = -0.348,  P = 0.295 | r_s_ = -0.048,  P = 0.888 | r_s_ = -0.329,  P = 0.324 | r_s_ = -0.078,  P = 0.819 |
| rs = 0.559,  P = 0.074 | rs = 0.417,  P = 0.202 | rs = 0.376,  P = 0.254 | rs = -0.14,  P = 0.681 | Total wet mass | r_s_ < 0.001,  P = 0.999 | r_s_ = 0.317,  P = 0.343 | r_s_ = 0.166,  P = 0.625 | **r_s_ = 0.832,**  **P = 0.001 *** | r_s_ = -0.383,  P = 0.245 |
| rs = 0.142,  P = 0.677 | rs = -0.161,  P = 0.637 | rs = -0.073,  P = 0.831 | rs = 0.435,  P = 0.182 | rs < 0.001,  P = 0.990 | Flight Metabolic Rate | r_s_ = 0.368,  P = 0.265 | **r_s_ = -0.602,**  **P = 0.05 *** | r_s_ = 0.172,  P = 0.613 | r_s_ = 0.269,  P = 0.423 |
| rs = 0.468,  P = 0.147 | rs = 0.45,  P = 0.165 | rs = 0.193,  P = 0.569 | rs = -0.348,  P = 0.295 | rs = 0.317,  P = 0.343 | rs = -0.368,  P = 0.265 | Forewing Length | r_s_ = 0.546,  P = 0.083 | r_s_ = 0.099,  P = 0.771 | r_s_ = -0.265,  P = 0.43 |
| rs = -0.117,  P = 0.731 | rs = -0.033,  P = 0.924 | rs = -0.307,  P = 0.358 | rs = -0.048,  P = 0.888 | rs = 0.166,  P = 0.625 | **rs = -0.602,**  **P = 0.05 *** | rs = 0.546, P = 0.083 | Wing Loading | r_s_ = -0.234,  P = 0.489 | r_s_ = -0.334,  P = 0.315 |
| rs = 0.554,  P = 0.077 | rs = 0.457,  P = 0.157 | rs = 0.286,  P = 0.393 | rs = -0.329,  P = 0.324 | **rs = 0.832,**  **P = 0.001 *** | rs = 0.172,  P = 0.613 | rs = 0.099,  P = 0.771 | rs = -0.234,  P = 0.489 | Wing Aspect Ratio | r_s_ = -0.36,  P = 0.276 |
| rs = -0.187,  P = 0.581 | rs = -0.2,  P = 0.556 | rs = 0.12,  P = 0.973 | rs = -0.078,  P = 0.819 | rs = -0.383,  P = 0.245 | rs = 0.269,  P = 0.423 | rs = -0.265,  P = 0.43 | rs = -0.334,  P = 0.315 | rs = -0.36,  P = 0.276 | Thorax-Abdomen Ratio |

**Table S1** Results of correlations (cor.test) between physiological, morphological and flight parameters in the core population of *Pieris* *mannii*. Significant P-values (P < 0.05) are shown in bold

**Table S2** Results of correlations (cor.test) between physiological, morphological and flight parameters in the edge population of *Pieris mannii*. Significant P-values (P < 0.05) are shown in bold

| Flight Duration | **r_s_ = 0.981,**  **P < 0.001 *** | r_s_ = 0.326,  P = 0.357 | r_s_ = 0.421,  P = 0.225 | **r_s_ = -0.699,**  **P = 0.024 *** | **r_s_** = 0.503,  P = 0.138 | **r_s_** = -0.353,  P = 0.318 | **r_s_** = -0.173,  P = 0.633 | **r_s_ = 0.636,**  **P = 0.048 *** | **r_s_** = 0.111,  P = 0.76 |
| --- | --- | --- | --- | --- | --- | --- | --- | --- | --- |
| **rs = 0.981,**  **P < 0.001 *** | Flight Distance | **r_s_** = 0.193,  P = 0.592 | **r_s_** = 0.399, P  = 0.254 | **r_s_ = 0.739,**  **P = 0.015 *** | **r_s_** = 0.556,  P = 0.095 | **r_s_** = -0.302,  P = 0.397 | **r_s_** = -0.136,  P = 0.707 | **r_s_** = 0.55,  P = 0.099 | **r_s_** = 0.063,  P = 0.862 |
| rs = 0.326,  P = 0.357 | rs = 0.193,  P = 0.592 | Maximal Velocity | **r_s_** = -0.124,  P = 0.733 | **r_s_** = 0.399,  P = 0.254 | **r_s_** = -0.348,  P = 0.325 | **r_s_** = -0.007,  P = 0.984 | **r_s_** = -0.267,  P = 0.457 | **r_s_** = 0.602,  P = 0.065 | **r_s_** = 0.069,  P = 0.849 |
| rs = 0.421,  P = 0.225 | rs = 0.399,  P = 0.254 | rs = -0.164,  P = 0.651 | Flight endurance | **r_s_** = 0.297,  P = 0.405 | **r_s_** = 0.672,  P = 0.033 | **r_s_** = -0.124,  P = 0.733 | **r_s_** = -0.104,  P = 0.775 | **r_s_** = -0.104,  P = 0.775 | **r_s_** = 0.289,  P = 0.418 |
| **rs = -0.699,**  **P = 0.024 *** | rs = 0.739,  P = 0.015 | rs = -0.124,  P = 0.733 | rs = 0.297,  P = 0.405 | Total wet mass | **r_s_** = 0.415,  P = 0.234 | **r_s_** = -0.23,  P = 0.523 | **r_s_** = 0.041,  P = 0.91 | **r_s_** = 0.148,  P = 0.683 | **r_s_** = 0.369,  P = 0.294 |
| rs = 0.503,  P = 0.138 | rs = 0.556,  P = 0.095 | rs = -0.348,  P = 0.325 | rs = 0.672,  P = 0.033 | rs = 0.415,  P = 0.234 | Flight metabolic Rate | **r_s_** = -0.175,  P = 0.638 | **r_s_** = -0.498,  P = 0.143 | **r_s_** = -0.048,  P = 0.896 | **r_s_** = 0.189,  P = 0.602 |
| rs = -0.353,  P = 0.318 | rs = -0.302,  P = 0.397 | rs = -0.007,  P = 0.984 | rs = -0.056,  P = 0.878 | rs = -0.23,  P = 0.523 | rs = -0.175,  P = 0.638 | Forewing Length | **r_s_** = -0.14,  P = 0.699 | **r_s_** = -0.536,  P = 0.11 | **r_s_** = 0.383,  P = 0.274 |
| rs = -0.173,  P = 0.633 | rs = -0.136,  P = 0.707 | rs = -0.267,  P = 0.457 | rs = -0.124,  P = 0.733 | rs = 0.041,  P = 0.91 | rs = -0.498,  P = 0.143 | rs = -0.14,  P = 0.699 | Wing Loading | **r_s_** = -0.041,  P = 0.91 | **r_s_** = -0.469,  P = 0.172 |
| **rs = 0.636,**  **P = 0.048 *** | rs = 0.55,  P = 0.099 | rs = 0.602,  P = 0.065 | rs = -0.104,  P = 0.775 | rs = 0.148,  P = 0.683 | rs = -0.048,  P = 0.896 | rs = -0.536,  P = 0.11 | rs = -0.041,  P = 0.91 | Wing Aspect Ratio | **r_s_** = -0.141,  P = 0.699 |
| rs = 0.111,  P = 0.76 | rs = 0.063,  P = 0.862 | rs = 0.069,  P = 0.849 | rs = 0.289,  P = 0.418 | rs = 0.369,  P = 0.294 | rs = 0.189,  P = 0.602 | rs = 0.383,  P = 0.274 | rs = -0.469,  P = 0.172 | rs = -0.141,  P = 0.699 | Thorax-Abdomen Ratio |
